# Microbial diversity as the next frontier of payments for ecosystem services in nature-positive finance

**DOI:** 10.1038/s44185-026-00136-7

**Published:** 2026-05-18

**Authors:** Kohei Ito, Honami Ando, Hiroshi Honda

**Affiliations:** 1 BIOTA Inc., Tokyo, Japan; 2 Keio Research Institute at SFC, Keio University, Kanagawa, Japan; 3 Honda Biotech. Laboratory, Tochigi, Japan; 4 University of Yamanashi, Yamanashi, Japan

**Keywords:** Ecology, Ecology, Environmental sciences, Microbiology

## Abstract

Payments for Ecosystem Services (PES) have matured across forests (carbon), watersheds (water quality and quantity), agricultural landscapes (biodiversity), and urban stormwater management. Most biodiversity assessments emphasize insects, fish, and birds. By contrast, schemes that directly measure and reward the microbial diversity, the foundations of ecosystem services in soils, aquatic systems, and urban green spaces, remain scarce. Meanwhile, the Kunming-Montreal Global Biodiversity Framework calls for mobilizing at least USD 200 billion per year by 2030, and the Global Biodiversity Framework Fund has launched; but current flows into PES are limited, and PES explicitly targeting microbes is largely uncharted. This Perspective argues that microbially focused PES is rare and, even when they exist, they tend to be biased toward specific pathogens or pollution indicators. However, measurement, reporting, and verification of various microorganisms are operationally feasible and methods to strengthen source attribution and contribution estimation are now mature; and channeling a share of Nature-Positive finance to microbial ecosystem assessments could jointly advance drinking-water safety, soil health, agricultural resilience, and urban public health. We discuss the importance of microbial-PES in conserving and enhancing ecosystem services.

## Introduction

Driven by the global push toward “Nature Positive,” the importance of biodiversity assessment is rising. The Kunming-Montreal Global Biodiversity Framework (GBF) asks Parties to mobilize at least USD 200 billion per year from all sources by 2030, including USD 30 billion per year in international finance^1^, and the Global Biodiversity Framework Fund (GBFF) has been launched, reaching pledges of roughly USD 386 million by mid-2025^2^. In parallel, corporations are beginning to disclose nature-related interfaces and risks through frameworks such as the Taskforce on Nature-related Financial Disclosures (TNFD), which provides a risk management and disclosure framework for organizations to report and act on evolving nature-related issues^3^.

Payments for Ecosystem Services (PES) is an outcome-oriented mechanism in which beneficiaries (e.g., utilities, municipalities, developers, consumers) make conditional payments to ecosystem-service providers (e.g., landowners, communities, protected-area managers)^4^. Typical examples include water tariffs financing upstream forest conservation^5^, premium pricing for biodiversity-friendly agriculture^6^, and carbon finance funding mangrove protection^7^. These well-known PES cases demonstrate effectiveness, and integrating PES with Nature-Positive capital could further stabilize service provision.

**Fig. 1:**
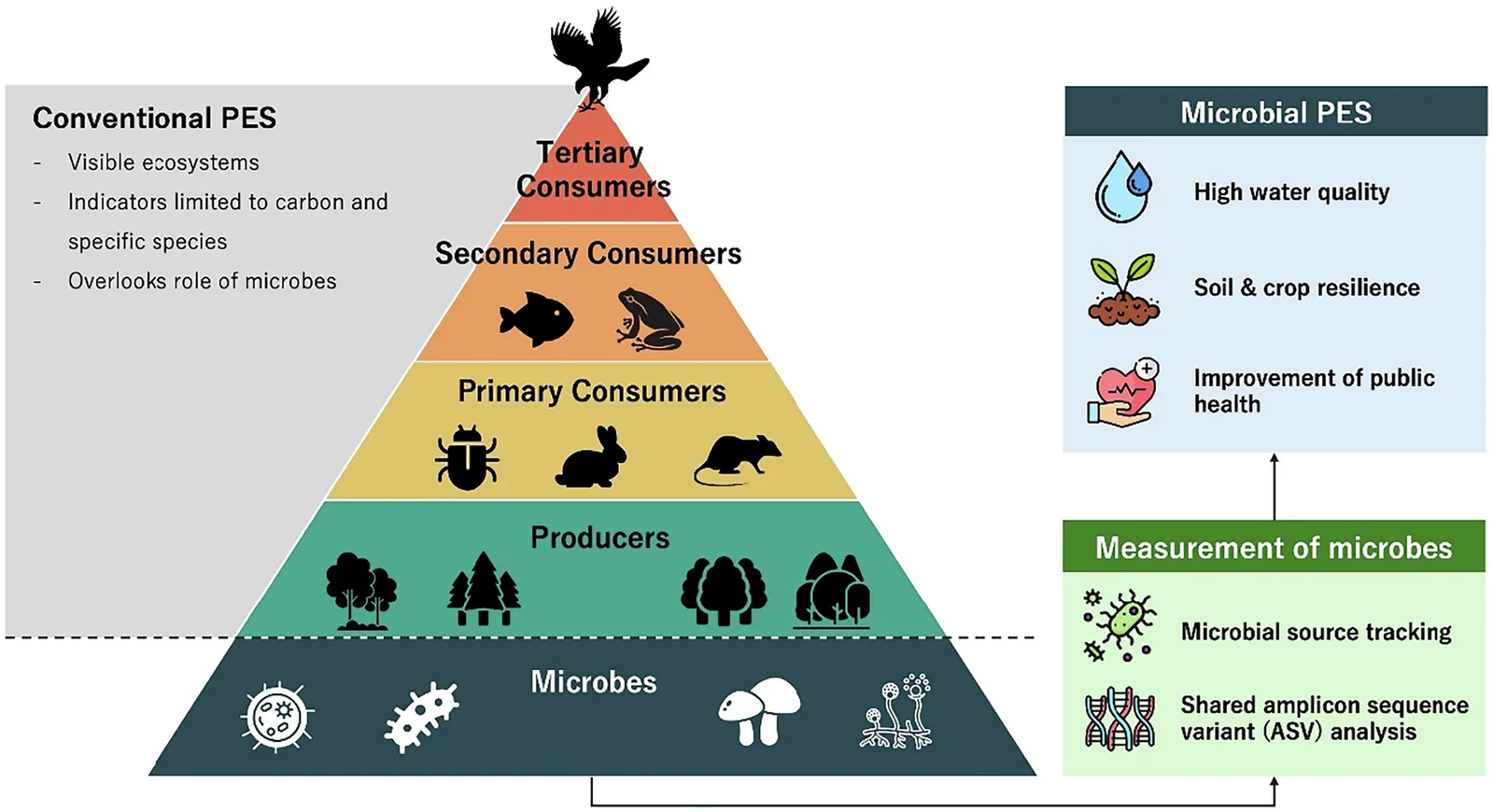
Integrating Microbial Diversity into PES: From Pathogen Control to Health Promotion. Microbes sustain the entire ecological pyramid through their multifunctional roles. Beyond serving as decomposers that drive nutrient recycling, they encompass phototrophs contributing to primary production and carbon sequestration (relevant to carbon credit systems), as well as parasites that directly influence public health. This conceptual diagram illustrates how evaluating microbial diversity at the foundation of the ecological pyramid improves outcomes across ecosystem services and human well-being. While traditional frameworks focus on visible biota and mitigating sanitary risks (e.g., pathogens/ fecal indicator bacteria (FIB)), integrating microbial community metrics captures the foundational drivers of ecosystem services. This approach enables a transition from reactive “defensive” risk management to proactive “offensive” health promotion and resilience.

However, relying solely on visible taxa and habitats—forests, wetlands, birds, fish—fails to capture the foundational mechanisms linking biodiversity, ecosystem services, and human health. This macro-centric view overlooks the “invisible biota” that underpin virtually all life-support systems: microbial diversity. Microbes inhabit every environment on Earth, from natural ecosystems to urban settings, and would drive carbon and nitrogen cycling, water purification, soil fertility, agricultural resilience, human health regulation, and even verification of food characteristics, safety, and quality (Fig. 1). Elevating microbial diversity to outcomes within PES would value foundational functions long overlooked and enable durable finance for natural capital. This Perspective reviews current PES targets, examines the opportunity to center microbes, and sketches institutional designs that braid Nature-Positive finance with microbial-PES.

## What does pes currently pay for?

PES schemes worldwide have largely targeted biota and services that are visible and socially legible (Table 1). In forests, carbon sequestration by tree communities and emblematic biodiversity (forest birds, large mammals) takes centre stage, as in Costa Rica’s national PES (financed by a fuel tax since 1997) and Mexico’s PSAH (Programa de Servicios Ambientales Hidrológicos) for hydrological services^8,9^.

**Table 1:** Representative PES cases by ecosystem type, focal biota, key services, and examples. FAD: filtration avoidance determination

Ecosystem type	Focal biota most often targeted	Key services/outcome metrics	Representative PES cases
Forests (temperate/tropical)	Tree communities; forest birds; large mammals	Carbon sequestration & storage; water regulation; erosion control; biodiversity	Costa Rica national PES (FONAFIFO)^8^; Mexico PSAH (hydrological services)^8,9^
Watersheds/drinking-water sources (rivers, lakes, aquifers)	Riparian vegetation; aquatic insects; freshwater fish; wetland birds	Drinking-water quality; baseflow/quantity; sediment control	NYC Catskill–Delaware watershed (FAD) ; Quito Water Fund, FONAG; Vittel (France) user-financed PES^5,10^
Agro-ecosystems (paddy landscapes, grasslands)	Paddy fauna (dragonflies, frogs); farmland birds; grassland flora	Farmland biodiversity; flood mitigation (paddy retention); cultural landscape	Sado “Crested Ibis–certified rice” (Japan); Toyooka “Stork-friendly farming” (Japan); EU agri-environmental eco-schemes^11,12^
Wetlands & mangroves (blue carbon)	Mangrove forests; benthic invertebrates, shorebirds	Blue-carbon storage; coastal protection; water purification; nursery habitat	Mikoko Pamoja (Kenya)^13^
Urban ecosystems/green infrastructure	Street trees, rain gardens, green roofs; urban birds & insects	Stormwater retention & water quality; urban heat mitigation; amenity	Washington, DC Stormwater Retention Credit market^14^; Philadelphia stormwater grants/credits^15^
Marine & coastal (reefs, seagrass)	Coral reefs; seagrasses; herbivorous fish; sea turtles	Tourism & recreation; shoreline protection; sustainable fisheries	Bonaire National Marine Park user-fee system^16^

In watersheds, outcomes include turbidity, nutrients, and drinking-water safety. The New York City Catskills program is emblematic: rather than build a costly filtration plant, NYC pays for upstream land-management improvements under the Filtration Avoidance Determination (FAD)^5^. Water funds such as the Fund for the Protection of Water (FONAG) in Quito and Vittel’s source-water protection are likewise canonical^10^. In agricultural landscapes, iconic rice-paddy fauna (dragonflies, frogs) and farmland birds feature prominently; Japan’s Toki- and Stork-friendly farming are well known^11^, and EU agri-environment schemes (AES) support biodiversity via direct payments to farmers who implement environmental management practices on their land^12^. In wetlands and mangroves, attention centers on mangrove forests, benthic fauna, and migratory birds; Kenya’s Mikoko Pamoja—the first community-led blue-carbon credit project—has become internationally influential^13^. Urban programs link trees, rain gardens, and green roofs to stormwater retention and heat-island mitigation; Washington, DC’s Stormwater Retention Credit market^14^ and Philadelphia’s crediting scheme monetize retention performance^15^. Marine PES analogs focus on coral reefs, seagrass, turtles, and herbivorous fish, with user-fee models in Bonaire^16^.

Across these cases, target biota cluster around trees, farmland flagship species, riverine and wetland fish and waterbirds, coastal corals and seagrasses, and urban birds and insects—i.e., high-visibility assemblages. Indicator sets likewise emphasize simplified metrics: tons of carbon, forest cover, turbidity, nutrients, runoff volume, and amenity value. By contrast, linking microbial diversity and function in soils and waters directly to payments remains rare. Consequently, current frameworks tend to address microbes solely as risks to be managed (e.g., pathogens), overlooking the opportunity to leverage diverse microbiomes for proactive health promotion. If scientifically grounded indicator species, including those from microbial communities, can be identified, monitored, and translated into measurable metrics, PES schemes have the potential to evolve from reactive risk management to instruments that actively advance both ecosystem integrity and human health.

## The current state of “microbial PES”

Where PES (or cognate schemes) engage microbes, evaluation metrics overwhelmingly prioritize drinking-water safety and sanitary risk via pathogens and fecal indicator bacteria (FIB), with few cases that explicitly include community-level diversity or functional metrics (Table 2). Even in flagship programs like NYC’s^5^, performance metrics are strictly limited to specific pathogens and FIB, excluding community-level diversity or functions. In Bolivia’s Watershared, a randomized controlled trial was used to evaluate whether incentives for land-use improvements could increase microbial water quality; however, evaluation still centers on a single FIB (*E. coli*)^17^. Latin American Water Funds (e.g., FONAG; FORAGUA) route city and corporate finance to upstream conservation with outcomes in flow and water quality, but microbial diversity and functions remain outside contractual metrics^10,18^. In cities, green stormwater infrastructure (GSI) (rain gardens and constructed wetlands) often tracks log reductions in FIB at outfalls, with performance varying by site and design; microbial diversity/function is monitored, if at all, as a supplementary indicator rather than an outcome^19,20^. Overall, current “microbe × PES” framework functions solely from the perspective of mitigating the negative impacts of specific microbes.

**Table 2:** PES schemes involving microbial outcomes: evaluation metrics, microbial focus, and limitations. FIB: fecal indicator bacteria

Scheme/Region	Outcome metrics	Microbial focus	Monitoring methods	Payment linkage	Limitations
NYC Catskill–Delaware watershed (USA)^5^	Drinking-water safety, FAD maintenance	FIB/pathogens (*E. coli*, *Giardia*, *Cryptosporidium*)	Regulatory monitoring, land-management compliance	Payments for upstream land management	Microbial diversity and functions not included
Bolivia “Watershared” program^17^	Reduction in *E. coli* (validated by RCT)	FIB only (*E. coli*)	Water sampling in RCT design	Payments to upstream landholders	Diversity and functions excluded
Water Funds in Latin America (e.g., Quito FONAG, FORAGUA)^10^	Water quality and quantity	FIB/general microbial contamination	Routine water monitoring	User contributions to upstream conservation	Diversity rarely included
Urban GSI (constructed wetlands, bioretention, rain gardens)^19,20^	FIB log reduction at outflow	FIB indicators (*E. coli*, Enterococci)	On-site sampling, performance evaluation	Stormwater fee credits, grants	High variability; diversity remains supplementary

Microbial diversity and functional diversity, however, have been associated with health-relevant outcomes^21,22^. Contact with natural environments alters the human microbiome, supports immune development, and is thought to reduce the risk of immune-related diseases^23^. Thus, in urban development, attention should not be limited to visible biological communities; maintaining and conserving native microbial assemblages is considered important for human health care. This aligns with the “biodiversity hypothesis,” which suggests that contact with diverse environmental microbiota regulates immune function and reduces the risk of non-communicable diseases^24^. However, current PES frameworks remain reactive, focusing almost exclusively on “defense” (mitigating pathogen risks) while ignoring the potential for “offense” (actively promoting immune resilience through exposure to microbial diversity). Consequently, frameworks that explicitly value and pay for these health-promoting microbial functions are virtually nonexistent.

## Why extend PES to microbes?

Current PES targets visible ecosystems and macro-biota, and relies on carbon, hydrologic metrics, or minimal sanitary indicators (e.g. FIB). As the ubiquitous engines of biogeochemical cycles, microbial communities in soils and waters drive the regulation, provisioning, and supporting services that sustain human well-being. Therefore, extending PES to these “invisible engines” is a necessary step toward capturing function and resilience more directly. To operationalize this shift, it is essential to distinguish between the microbial-PES framework itself and its operational components. While microbial diversity and functions represent the desired ecological outcomes (e.g., resilience and nutrient cycling), microbial diversity metrics (such as amplicon sequence variants or functional gene abundances) serve as the measurable units for reporting. Furthermore, technologies like microbial source attribution provide the necessary verification tools to link these metrics to specific management actions.

In conventional PES, the role of biodiversity is context-dependent, serving as a proxy, target, or mechanism depending on the evaluation framework (Supplementary Table S1). Similarly, in microbial-PES, microbial diversity can act as an indirect indicator of ecosystem function, while also being treated as a direct target under Nature-Positive goals, thereby linking biodiversity conservation with functional outcomes. The indirect indicator in microbial-PES may be treated as targets where their functional/quality relevance and underlying mechanisms are sufficiently clarified. Furthermore, since microbial communities exhibit strong spatial heterogeneity, making standardized or universally applicable optimization difficult. Therefore, we consider that adopting microbial diversity as a primary indicator represents a meaningful and pragmatic first step in the development of microbial-PES frameworks.

The potential applications of microbial PES are as follows. First, microbial diversity underpins ecosystem stability. Diverse soil communities conduct decomposition and nitrogen cycling; higher diversity buffers function under stressors such as drought and temperature variability^25^. This stability will elevate agricultural productivity and watershed retention while reducing erosion and sustaining long-term carbon storage, bringing the “invisible foundation” of soils into scope for PES. Second, adding microbial diversity metrics to source water and distribution systems can refine drinking-water safety assessment. While a single FIB monitoring provides minimum safeguards but remains insufficient for water safety^26^, microbial diversity-based monitoring would be a promising viewpoint for health and early anomalies^27^, moving payments beyond “compliance” to durable guarantees of water quality. Third, soil microbes link directly to carbon sequestration and agricultural resilience. Through stabilization of soil organic carbon and aggregation, microbes would facilitate carbon storage, nutrient provisioning, and disease resistance—jointly benefiting climate mitigation and food security^28^. Fourth, credible measurement, reporting, and verification (MRV) is essential. Technologies like Microbial Source Tracking (MST)^29^ and shared Amplicon Sequence Variant (ASV) analysis^30,31^ now enable precise causal attribution and the tracking of functional linkages across environments. Integrated with traditional pathogen indicators, these methods allow PES to evidence both risk reduction and functional recovery. However, advancing microbial-PES requires systematic meta-comparison of microbial diversity across ecosystems, together with refined identification of taxa most relevant to water quality and public health. Consolidating this knowledge base is crucial to operationalize microbial indicators as reliable outcome metrics for PES schemes. Finally, extending microbial PES in urban development can couple environmental and public-health goals. Green infrastructure can reduce FIB during storm events^32^ while enriching human exposure to diverse environmental microbiota, with potential benefits for allergy and autoimmune risk reduction^23,33^. In recent years, the idea of using “Microbiome rewilding” to address public health issues in cities has been proposed^34^. This concept views green infrastructure as a source of microbes. In this sense, urban PES has the potential to evolve into a mechanism that simultaneously “purchases” both conventional sanitary protection and preventive health promotion. Adopting this perspective would enable investments by municipalities and corporations to simultaneously realize dual benefits in environmental improvement and public-health enhancement. To advance this framework, epidemiological investigations are required to clarify how the spatiotemporal distribution of urban microbial communities relates to patterns of human disease incidence, and to define appropriate forms of microbial exposure in terms of both quantity and quality.

## Nature-positive finance: Where is it flowing?

In recent years, propelled by the “Nature Positive” agenda and GBF targets, private investment in biodiversity and natural capital has surged. UNEP FI reports nature-related finance exceeded USD 100 billion in 2024, growing over 10-fold since 2020^35^. Nevertheless, achieving the GBF’s 2030 targets will still require additional investment, and a substantial funding gap persists. Within these flows, capital that directly links to PES remains only a small fraction of the total. Syntheses of nature-based solutions finance suggest that private finance flowing specifically into PES schemes is low single-digit billions^35–37^, i.e. a minor share of total nature-related flows. Recent PES programs heavily rely on public and donor funding, underscoring their currently peripheral role within the broader landscape of nature finance^38^.

By sector, the largest volumes are directed to forest restoration, coastal and marine conservation, and sustainable agro-forestry projects^39^. These activities align with Nature-Positive objectives, but their performance indicators tend to privilege visible outputs such as hectares planted or expansion of protected areas. By contrast, initiatives that set ecosystem services themselves—for example, water-resources management or improvements to agricultural landscapes—as contracted outcomes remain smaller in scale, and long-term, performance-linked PES designs are still in the minority^40^.

As highlighted by the OECD, institutional constraints also inhibit the scaling of PES, as it emphasizes that expanding biodiversity-positive subsidies and PES is essential, emphasizing that standardized and transparent MRV of outcomes is key to mobilizing private capital^37^. In addition, new policy frameworks are emerging, such as the European Union’s consideration of “nature credits”^41^, and these may enable the redirection of nature-related investment into PES structures^42^. Accordingly, we argue that explicit consideration of microbial diversity is an important component in the development and implementation of nature credits. Integrating microbial diversity metrics may provide a pathway to better inform Nature-Positive finance by making otherwise “hidden” risks, such as emerging pathogen dynamics or gradual soil degradation, more detectable. In this sense, microbial indicators could contribute to improving risk assessment and long-term environmental performance evaluation. At the same time, the extent to which such indicators can be incorporated into investment decision-making will depend on the accumulation of robust causal evidence linking microbial diversity dynamics to ecosystem-service outcomes. The MST and ASV sharing offer promising tools in this regard, but further empirical investigations are required.

Importantly, the translation of microbial indicators into financial flows is also a question of governance. The adoption and standardization of indicators within MRV systems, as well as their integration into crediting or PES schemes, will likely depend on institutional actors. In particular, early recognition of the potential value of microbial diversity by public authorities and international frameworks may play a key role in shaping standards and guiding capital allocation. At the same time, effective integration with private finance and market-based instruments will be essential. Thus, rather than directly transforming investment flows, microbial-inclusive PES may provide an enabling framework through which ecological knowledge, policy design, and financial mechanisms can become more closely aligned.

## Conclusion

We reviewed the state of PES, highlighting the paucity of frameworks that target microbial communities. Existing programs focus on visible ecosystems, with indicators centered on carbon and FIB. However, bringing microbial diversity and functions into outcome sets would be essential to assess resilience and public value accurately. In water systems, community metrics can sharpen safety assurance; in soils and agriculture, they can tie payments to carbon sequestration, fertilizer efficiency, and drought tolerance; and in cities, green infrastructure can enrich environmental microbiota alongside sanitary gains (Fig. 1). Robust MRV can quantify sources and causal links, making dual benefits (“risk reduction” plus “functional recovery”) visible and auditable. Although Nature-Positive capital is expanding, PES-linked flows are still limited. Embedding microbially grounded outcomes can redirect investment from solely visible assets to the invisible foundations. This shift allows PES to function as a comprehensive health investment instrument, integrating benefits across water safety, agricultural resilience, and human immune health.

## Supplementary information

Supplementary information

## Data Availability

Not applicable.
