# Supplementary material for: Microbial diversity as the next frontier of payments for ecosystem services in nature-positive finance
Source: NPJ Biodivers. 2026 May 18;5:27. doi: 10.1038/s44185-026-00136-7 (PMC13590658; doi:10.1038/s44185-026-00136-7)
Supplement: Supplementary file 1 [file 44185_2026_136_MOESM1_ESM.pdf]

Ito *et al*. 2026, Microbial Diversity as the Next Frontier of Payments for Ecosystem Services in Nature-Positive Finance, npj biodiversity

Supplementary Table S1. Context-dependent and non-exclusive roles of diversity in conventional and microbial PES frameworks

Ecosystem service / outcome	Conventional PES example	Typical indicator in conventional PES	Role of diversity in conventional PES	Role of microbial diversity in microbial-PES (function perspective)	Role of microbial diversity in microbial-PES (Nature-Positive perspective)
Water safety	NYC Catskills watershed PES	FIB (E. coli), turbidity, pathogen compliance	Proxy (ecosystem condition inferred from water quality indicators)	Proxy* / Mechanism (diversity reflects system stability and contributes to pathogen suppression)	Target (maintaining microbial diversity as part of aquatic biodiversity)
Carbon sequestration	Mangrove PES (e.g., Mikoko Pamoja)	Carbon stock, biomass, soil carbon	Mechanism / Proxy (vegetation drives carbon storage; biodiversity often indirect)	Mechanism / Proxy* (microbes regulate carbon stabilization; diversity indicates functional capacity)	Target (soil and sediment microbial diversity as part of biodiversity conservation)
Nutrient cycling / agriculture	Toki-friendly rice (Japan), agri-environment schemes	Indicator species (e.g., ibis), reduced agrochemical input	Proxy / Target (biodiversity used as proxy; sometimes explicit conservation target)	Mechanism / Proxy* (microbial diversity underpins nutrient cycling and soil fertility)	Target (soil microbial diversity as biodiversity outcome under Nature-Positive goals)
Pathogen suppression	Water quality PES / sanitation programs	Reduction of pathogens or FIB	Proxy/Target (indicator organisms reflect sanitary condition)	Mechanism / Proxy* (community interactions suppress pathogens; diversity signals system state)	Target (limited) (less commonly explicit, but may be included under biodiversity framing)
Immune regulation / public health	Urban green infrastructure (emerging PES domain)	Health outcomes, exposure metrics	Mechanism (implicit) (biodiversity linked to health but rarely formalized)	Mechanism (microbial exposure influences immune development)	Target (microbial diversity as beneficial exposure and public health asset)

* Proxies in microbial-PES may be treated as targets where their functional relevance and underlying mechanisms are sufficiently clarified.
